# Cross-Modality Imaging of Murine Tumor Vasculature—a Feasibility Study

**DOI:** 10.1007/s11307-021-01615-y

**Published:** 2021-06-08

**Authors:** Lydia M. Zopf, Patrick Heimel, Stefan H. Geyer, Anoop Kavirayani, Susanne Reier, Vanessa Fröhlich, Alexander Stiglbauer-Tscholakoff, Zhe Chen, Lukas Nics, Jelena Zinnanti, Wolfgang Drexler, Markus Mitterhauser, Thomas Helbich, Wolfgang J. Weninger, Paul Slezak, Anna Obenauf, Katja Bühler, Andreas Walter

**Affiliations:** 1 Austrian BioImaging/CMI, Vienna BioCenter Core Facilities GmbH (VBCF), Vienna, Austria; 2 Ludwig Boltzmann Institute for Experimental and Clinical Traumatology in the AUVA Trauma Research Center, Austrian BioImaging/CMI, Vienna, Austria; 3 Division of Anatomy, MIC, Medical University of Vienna, Austrian BioImaging/CMI, Vienna, Austria; 4 Department of Biomedical Imaging and Image-guided Therapy, Division of Molecular and Structural Preclinical Imaging, Medical University of Vienna, Vienna, Austria; 5 Medical University of Vienna, Vienna, Austria; 6 Research Institute of Molecular Pathology (IMP), Vienna Biocenter (VBC), Vienna, Austria; 7 VRVis Zentrum für Virtual Reality und Visualisierung Forschungs-GmbH, Austrian BioImaging/CMI, Vienna, Austria; 8 Ludwig Boltzmann Institute Applied Diagnostics, Vienna, Austria; 9 Core Facility Hard Tissue and Biomaterial Research, Karl Donath Laboratory, University Clinic of Dentistry, Medical University Vienna, Vienna, Austria

**Keywords:** Mulitmodal imaging, Correlative imaging, Bioimaging, Tumor vasculature, Angiogenesis, Acquired resistance, Preclinical imaging

## Abstract

Tumor vasculature and angiogenesis play a crucial role in tumor progression. Their visualization is therefore of utmost importance to the community. In this proof-of-principle study, we have established a novel cross-modality imaging (CMI) pipeline to characterize exactly the same murine tumors across scales and penetration depths, using orthotopic models of melanoma cancer. This allowed the acquisition of a comprehensive set of vascular parameters for a single tumor. The workflow visualizes capillaries at different length scales, puts them into the context of the overall tumor vessel network and allows quantification and comparison of vessel densities and morphologies by different modalities. The workflow adds information about hypoxia and blood flow rates. The CMI approach includes well-established technologies such as magnetic resonance imaging (MRI), positron emission tomography (PET), computed tomography (CT), and ultrasound (US), and modalities that are recent entrants into preclinical discovery such as optical coherence tomography (OCT) and high-resolution episcopic microscopy (HREM). This novel CMI platform establishes the feasibility of combining these technologies using an extensive image processing pipeline. Despite the challenges pertaining to the integration of microscopic and macroscopic data across spatial resolutions, we also established an open-source pipeline for the semi-automated co-registration of the diverse multiscale datasets, which enables truly correlative vascular imaging. Although focused on tumor vasculature, our CMI platform can be used to tackle a multitude of research questions in cancer biology.

**Supplementary Information:**

The online version contains supplementary material available at 10.1007/s11307-021-01615-y.

## Introduction

Cancer is a leading cause of mortality worldwide. In Europe alone, 1 in 2 men and 1 in 3 women will be diagnosed with invasive cancer in their lifetime. In tumors, cancer cells are in constant interaction with the tumor stroma, immune cells, and the vasculature. The tumor vasculature plays a key role for cancer progression and dissemination. Blood vessels deliver nutrients and oxygen to support tumor growth, and angiogenesis, the sprouting of new blood vessels from the existing vasculature, is a hallmark of cancer and facilitates rapid metastasis [1–3]. Formation of new blood vessels can be triggered by hypoxia and ligands secreted by the tumor cells when the tumor surpasses the local supply of oxygen (among other triggers for the production of pro-angiogenic molecules, such as pH, mechanical stress, genetic alterations, or inflammatory responses) [4]. A current strategy for cancer treatment is the inhibition of neo-angiogenesis by anti-angiogenic therapies, which can result in tumor regression, as shown previously [5, 6]. However, the intricate interplay of vascular structure and function makes the inhibition of tumor angiogenesis more complex. Even though anti-angiogenic treatments initially reduce tumor growth, their success is limited due to intrinsic or acquired resistance to the drugs, resulting in increased leakiness of the vessels, tumor malignancy, and increased metastases [5–7]. Tumor vessels are disorganized, tortuous, often dilated, leaky, and dysfunctional, which interferes with the perfusion of drugs and infiltration of immune cells into the tumor [8, 9]. These morphological abnormalities do not seem to present obstacles to tumor growth but are likely to play a part in malignant progression [9]. Currently, many drugs fail to adequately target malignant tumor cells eventually leading to therapy failure. Improved targeting and more effective treatment strategies will require a better understanding of the tumor vasculature.

**Fig. 1:**
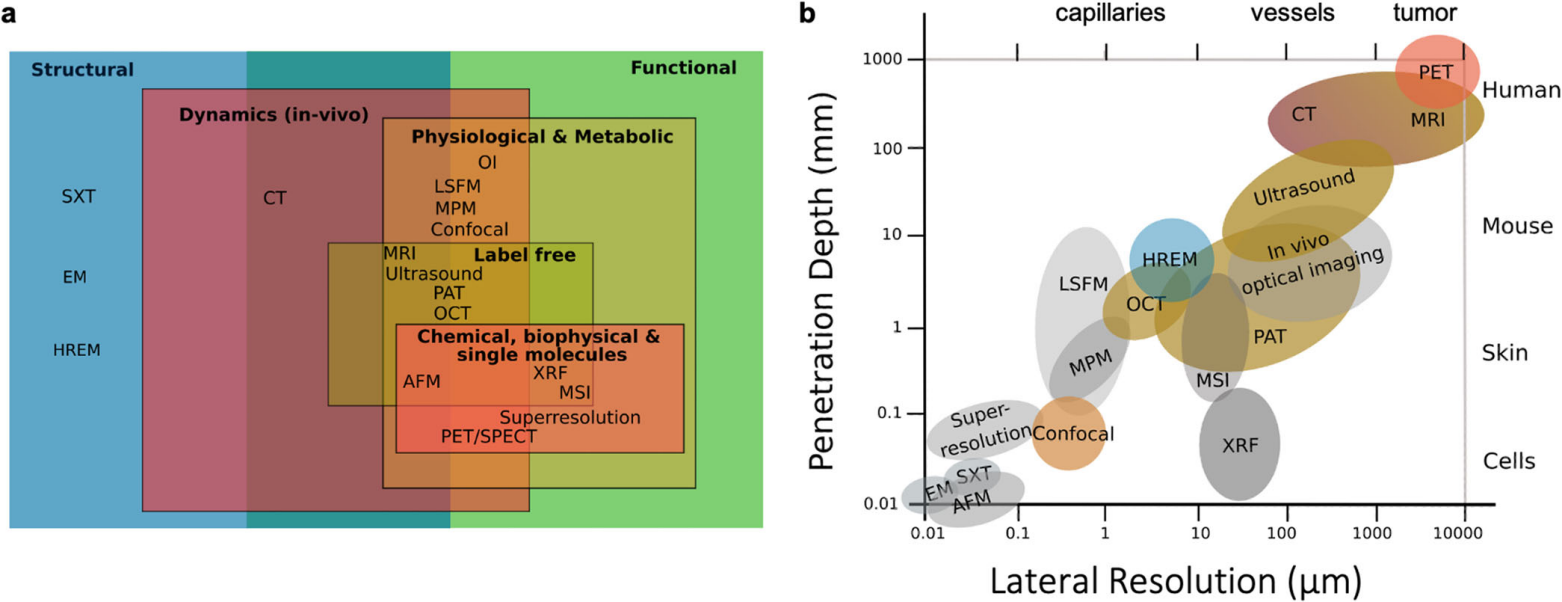
**a** Imaging modalities for assessing all relevant information scales for structural and functional imaging. **b** Accessible resolution to CMI. CMI is the only way to span 3D multiscale information across all relevant scales in biomedical research. Penetration depth refers to the maximal thickness the radiation can travel to yield a meaningful image or, in the case of HREM, to the volume that can be readily probed by the setup. Lateral resolution (in-plane resolution) is defined as the ability of the system to distinguish two points in the direction perpendicular to the direction of the illumination. It also depends on the depth of imaging. Modalities combined in this showcase are colored according to their information space (see a): EM (electron microscopy), SXT (soft X-ray tomography), AFM (atomic force microscopy), XRF (X-ray fluorescence spectrometry), superresolution (microscopy such as STORM, PALM, STED), confocal (microscopy), LSFM (light sheet fluorescence microscopy), MPM (multiphoton microscopy, OI (optical imaging–bioluminescence/fluorescence). Adapted from [18].

Over the past decade, imaging technologies have emerged that allow the examination of the tumor vasculature at high-resolution and molecular specificity, allowing researchers to assess for example, morphology, perfusion, and pH in living tissues with high precision [10–12]. Consequently, much of our understanding of the tumor vasculature and its role in cancer progression originates from preclinical studies that involve a variety of imaging modalities [13]. However, previously, specific features of the vasculature have mainly been studied independently. Correlating different parameters of the same tumor vasculature (i.e., from the same volumes of interest (VOIs)) using correlative and complementary imaging modalities across scales and their integration with functional insights remains mostly unchartered territory. Yet, the correlation of multiple vascular parameters is vital to gain holistic insights into tumor vascularization [14]. In biomedical imaging, it would be ideal to assess all relevant parameters from a single sample and maximize the information that can be obtained from it. This will allow correlation of all characteristics and gain a holistic understanding of the sample. Such a quantitative and exhaustive analysis of the sample can be achieved by cross-modality imaging (CMI). CMI combines two or more complementary modalities that create a highly informative, composite view of the sample and allows access to both structural and functional parameters across all relevant scales from the same sample. In contrast to the analysis of (non-identical) consecutive sections (as usually done in multimodality approaches); this enables true correlation and merging of data since a comprehensive parameter cloud can be acquired from almost the same timepoint for exactly the same VOI [15]. For tumor angiography specifically, it is desirable to visualize vascular networks from blood vessels to capillaries and correlate their structure with molecular, dynamic and functional parameters, such as blood flow or hypoxia, in the entire tumor. An example of a two-modality *ex vivo* study (SEM and CT) to correlate vascular morphology of regions of interest of a mouse brain and to image its vasculature at increasing resolution can be found in [16]. Tumor vasculature exhibits a large variety of vessel sizes, with the smallest capillaries measuring only a few microns in diameter [17]. Any single imaging modality is not sufficient to comprehensively capture the morphology and functional characteristics of the vasculature within the entire tumor at such high resolution since penetration depth comes at the expense of lateral resolution in most single imaging modalities (Fig. 1, Table 1). To achieve high-resolution imaging that can discern microvasculature, usually microscopy has to be applied. Microscopic imaging, however, usually needs to be applied *ex vivo* and does not exceed imaging depths of about 1 mm even for multiphoton microscopy—which is not enough to visualize the inner vascular network of the tumors that span beyond several millimeters in thickness [21]. Studying tumor vasculature thus commonly involves multiple imaging modalities.

**Table 1:** Imaging parameters, advantages, and limitations of the modalities employed in this study for visualizing tumor vasculature. Please compare with Fig. 2

Modality	Contrast	Pene-tration [mm]	Lateral resolution [μm]	VOI	Advantages	Limitations
microMRI	Endogenous ‘time-of-flight’ contrast based on the moving hydrogen nuclei inside blood vessels	> 500	≤ 100	Whole mouse	-Non-ionizing radiation -Very good soft tissue contrast -Biochemical information (spectroscopy)	-Expensive equipment -High maintenance costs -Minimum blood flow/velocity needed for MR angiography
US	Acoustic impedance between tissue interfaces; detection of echoes from moving scatterers, i.e., blood flow (Power Doppler)	< 150	30–800	Whole mouse	-High-temporal and high-spatial resolution -Portable instrumentation -Cost efficient -Non-ionizing	-Limited tissue penetration -Poor contrast -Difficult to quantitate
PAT	Acoustic waves generated by optical absorption of tissue chromophores (i.e., hemoglobin in blood)	~ 10	~ 40	10 × 10 mm^2^	-*In vivo* -Penetration depth -Endogenous and exogenous contrast	-Resolution -Speed -Structural contrast
OCT	Optical scattering based on refractive index changes;motion contrast due to blood flow (OCT angiography)	~ 1–2	1–10 (diffraction limited)	10 × 10 mm^2^	-*In vivo*-Fast -Non-invasive-Label-free-Morphology-quantitative blood flow	-Limited molecular information -Reduced subcellular resolution -Minimum blood flow required
microPET	Accumulation of radioactive agent [^18^F]FMISO in hypoxic areas due to an increased reductive milieu followed by conjugation with glutathione	> 500	1000–2000	Whole mouse	-High sensitivity -Fully quantitative -Broad range of applications (imaging agent dependent) -Dynamic measurements	-Use of radioactive agents -Highly specialized equipment and staff required -High costs
microCT	High X-ray attenuation of the vascular barium sulfate perfusion contrast agent compared to surrounding soft tissue	> 500	≤ 100	Whole mouse	-Excellent bone imaging	-Radiation dose -Low soft-tissue contrast (use of contrast agents for vasculature)
HREM	Light scattering based on unspecific eosin staining	Sample size up to 12 mm in thickness	> 1	8 mm × 8 mm × 12 mm	-Digital volumes in histologic quality at high resolution	-Whole mount contrasting of specimens -Time-consuming (fixation and acquisition of 3D volume) -*Ex vivo*, no dynamics, structural data only
HP	Light scattering (various staining methods impart color and contrast to cellular and tissue components)	< 0.1	> 0.3	Up to 1 mm^3^	-Evaluation of overall tissue features at low costs -Excellent cellular detail at light microscopic resolution -Spatial contextual correlation of microscopic morphology	-2D and static -Detailed evaluation (especially of abnormal features in lesions) requires additional expertise

This study goes beyond common multimodality approaches since we specifically target correlation and establish a workflow to image exactly the same tumor sequentially. Altogether, 8 modalities were used to study the tumor vasculature: Ultrahigh-field micro-magnetic resonance imaging (microMRI), micro-computed tomography (microCT), small-animal ultrasound (US), optical coherence, and photoacoustic tomography (OCT/PAT), micro-positron-emission tomography (microPET), high-resolution episcopic microscopy (HREM), and histopathology (HP). The combination of the imaging modalities aimed at (a) spanning relevant resolution ranges to characterize the vascular morphology both *in*- and *ex vivo* and (b) complementing the structure by the functional imaging parameters hypoxia and blood flow. This CMI approach integrates the advantages and strengths of each modality and creates synergistic benefits over any single modality since, for example, HREM and HP achieve the highest resolution but require irreversible *ex vivo* processing (e.g., sectioning) of the tumor; microMRI and microCT achieve relatively high *in vivo* resolutions within the overall tissue context but have low sensitivity; microPET is highly sensitive but has poor spatial resolution; and quantification of fluorescence or optoacoustic signals in thick tissues *in vivo* is limited due to aberration and attenuation by scattering and absorption. The general imaging parameters, contrast mechanisms, advantages, and limitations of the modalities of the CMI pipeline are summarized in Table 1. The CMI pipeline includes computational approaches to visualize and co-register the acquired multiscale datasets. We tackled this challenge by establishing a customized open-source co-registration pipeline for imaging data from microMRI, microCT, and HREM (excluding segmentation, which was only achieved using commercial software). The open-source aspect covered by the paper only refers to data co-registration, not to blood vessel segmentation. Importantly, the study is designed as a methodological proof-of-principle to showcase the feasibility and compatibility of such an extensive CMI (image acquisition) pipeline, including the co-registration of the vascular morphology across modalities from exactly the same VOI of the same tumor (open-source CMI software pipeline).

## Materials and Methods

### Cell Culture

The murine melanoma YUMM1.7 (*Braf*^V600E/WT^*Pten*^−/−^*Cdkn2a*^−/−^) parental [19] and RAFi-resistant cell line derivatives were cultured in DMEM-F12 media with 10 % FBS, 2 mM l-glutamine and 100 IU/ml penicillin/streptomycin. RAFi-resistant cells were maintained on 100 nM RAFi (dabrafenib, Selleckchem). All cells were grown at 37 °C with 5% CO_2_ and regularly tested negative for mycoplasma contamination.

### *In Vivo* Studies

All experiments using animals were performed in accordance with our protocol approved by the Austrian Ministry (BMBWF-66.015/0009-V/3b/2019 or GZ: 340118/2017/25). For injections, 6–12-week-old male/female immune-competent B6(Cg)-Tyr^c-2J/^J mice were used. For subcutaneous tumor cell injections, mice were anesthetized using ketamine hydrochloride (100 mg/kg), xylazine (10 mg/kg), and acepromazine (3 mg/kg) or isoflurane and 10^6^ tumor cells were subcutaneously injected into the shaved flank in 50 μl of Matrigel/PBS (1:1) (Corning).

### MicroMRI

#### Sample Preparation and Data Acquisition

MicroMRI was performed on a 15.2 T Bruker system (Bruker BioSpec, Ettlingen Germany) with a 35-mm quadrature birdcage coil. Mice were continuously monitored for tumor occurrence by palpation. When tumors reached 20–70 mm^3^, mice were first scanned using microMRI. All animals were anesthetized with isoflurane (4% induction, maintenance with 1.5%). To visualize blood vessels, 3D fast imaging with a steady-state free precession (FISP) sequence with a gradient echo readout was used (repetition time (TR)/echo time (TE) = 5.7/2.85 ms, 30° flip angle, 30 × 30 × 10 mm^3^ field of view, 500 × 500 × 50 matrix size, 60 × 60 μm^2^ in-plane resolution, 200 μm slice thickness, number of experiments [NEX] = 16). The obtained resolution (see Table 3) was calculated based on the field of view divided by the number of frequency and phase encoding steps (the imaging matrix). Increasing the imaging matrix decreases the signal-to-noise ratio.

#### Post-processing

A maximum intensity projection was performed to visualize blood vessels in 3D. Semi-automated vessel segmentation was performed in Amira (V6.0 Thermo Fisher Scientific).

### Small-Animal US

#### Sample Preparation and Data Acquisition

Mice were kept under inhalant anesthesia (isoflurane 2% and O_2_ with 2 L/min) via a mask during the entire experiment. Body temperature, breathing frequency and ECG were monitored over the whole duration of the experiment. Mice were placed in supine position and scanned with a dedicated small animal Ultrasound System (Vevo2100, VisualSonics, Toronto, Canada) in a total scan time of 15 min. B-Mode images in 2D and 3D were acquired as well as Power Doppler images in 2D and 3D. Additional pulse-wave Doppler measurements of the tumor veins, i.e., velocity, were performed. A high-frequency MS550S ultrasound probe (center frequency 32 MHz, maximum frequency 55 MHz) was used to perform high-resolution US measurements with a pixel size of 37 × 37 μm^2^ (see Table 3). Due to a fully automated scan stage, the imaging step size (z-resolution) for the acquired 3D datasets is 0.076 mm. The overall duration of the experiment per mouse (i.e., induction of anesthesia, preparation, total scan time, and end of anesthesia) was about 30 min.

#### Post-Processing

Post-processing of 3D data was performed using the in-built Software (Vevo Lab 3.2 0). Acquired 3D data of tumor volume in Power Doppler Mode provide grey-scale data of tumor tissue and color-coded data of vessels (arteries and veins). The grey-scale data of the tumor tissue were automatically excluded via the automated in-built software, and the volume of the tumor vasculature was displayed as percentage relative to the total tumor volume (perfusion volume).

### OCT/PAT

#### Sample Preparation and Data Acquisition

A swept source OCT system was combined with an OCT and a PAT system in terms of both the hardware and software. A novel akinetic swept source was used in the OCT system. Due to the high phase-stability of the source, a phase-based and complex signal-based OCT angiography (OCTA) algorithm was developed without any hardware implementation. More details on the hybrid system can be found in Chen et al. [20]. The resolution of the PAT system (see Table 3) was assessed by measuring the edge spread function of a rectangle on a ‘1951 USAF resolution target’, a cover glass coated with different patterns of thin film.

#### Post-Processing

OCTA angiograms were generated by calculating the difference between consecutive B-scans at the same position. For OCTA, the essential step for the vascular segmentation is the calculation of the difference of sequential tomograms acquired at the same position on the skin. OCT provides the morphological information and this information includes the DC terms, which is the static tissue, and AC terms, which is the moving blood cells and liquid in the vasculature. By the calculation of the difference of the tomograms, DC terms can be minimized, and AC terms can be maximized, and the vascular information can be segmented. More steps are used for enhancing the vascular contrast, such as thresholding and Gaussian filter. PAT is a technique thatcan provide the absorption contrast of tissue. The high-energy pulsed laser illuminates the skin, and the main absorbers in the skin (melanin and hemoglobin) convert the light energy into a photoacoustic wave. Then, the photoacoustic wave is detected by a Fabry-Perot transducer in the PAT system. We used a novel Fabry-Perot interferometer (FPI) sensor to detect the acoustic waves generated by thermoelastic expansion. 3D images of the chromophores (i.e., hemoglobin) can be reconstructed by detecting the thickness changes of the FPI sensor. Therefore, PAT can provide the contrast of the melanin layer and hemoglobin in the vasculature inherently without any segmented algorithm. During the experiments, we chose the least pigmented samples. The image reconstruction algorithm of PAT is similar toultrasound imaging and includes back-propagation reconstructions, Fourier transforms, thresholding, etc.

### MicroPET/CT

#### Sample Preparation and Data Acquisition

Tumor-bearing mice were kept under anesthesia with a mixture of isoflurane (2%) and O_2_ (2 L/min) continuously administered via a nose cone during the entire experiment. During the whole timeframe of the experiment, animals were kept at a body temperature of 37 °C and physiological parameter such as breathing frequency was monitored. Anesthetized animals were positioned in the imaging chamber of the small animal Inveon PET/SPECT-CT scanner (Siemens Medical Solutions, Knoxville, TN). The radiotracer [^18^F]FMISO (10–15 MBq) was injected via the lateral tail vein and the acquisition of static microPET images was performed 2 h post-injection over 10 min. Additionally, each animal was imaged for 10 min using high-resolution small-animal cone-beam CT (Inveon PET/SPECT/CT), which was co-registered to the PET image and used as anatomical landmarks to draw 3D VOIs. In hypoxic tissue, [^18^F]-FMISO is reduced, bound covalently to intracellular macromolecules, and quantifies the amount of hypoxia in cancer cells (compare Table 1).

#### Post-Processing

microPET images were reconstructed with the OSEM3D/OP-MAP reconstruction algorithm (OSEM iterations 4, MAP iterations 18, target resolution 1.5 mm) with scatter and attenuation correction. MicroCT raw data were reconstructed with a Feldkamp algorithm using a Ramp filter followed by standard mouse beam-hardening correction (matrix size 1024 × 1024; effective pixel size 97.56 μm). The standard data correction protocol (normalization, attenuation, decay correction, and injection decay correction) was applied to the data. 3D VOIs were semi-automatically outlined on multiple planes of the microPET summation images and the fused microCT image using the biomedical imaging quantification software PMOD (version 3.8, Pmod Ltd., Zurich, Switzerland). An automatic transformation matrix was applied for the correct registration of PET/CT images.

### MicroCT

#### Sample Preparation and Data Acquisition

All microCT scans were performed using a SCANCO microCT 50 (SCANCO Medical AG, Brüttisellen, Switzerland). The whole animal was perfused with 30% Micropaque (Guerbet GmbH, Villepinte, France), a barium sulfate perfusion contrast agent mixed with 2% (w/v) procine gelatin (Sigma-Aldrich, St. Louis, MO, USA), immediately postmortem and placed in a 50-ml centrifuge tube for microCT scanning. Before application of the contrast agent, perfusion surgery was performed as described in Gage et al. [22]. For the fixation procedure, saline with 1000 IU heparin (Gilvasan Pharma GmbH, Vienna, Austria) and 4% formaldehyde (VWR International, Radnor, PA, USA) was used. A perfusion of saline was followed by a perfusion of formalin, followed by another perfusion of saline. Each perfusion step was performed manually via a syringe for approximately 5 min. After perfusion fixation, the contrast agent mixture (heated to 40–50 °C) was injected using a 20-ml syringe. To check the progress of the contrast agent, a small cut in the skin of the inner thigh was made to expose the arteria femoralis and the adjacent vein. The contrast agent mixture was injected slowly. The white contrast agent is visible in vessels in the skin and in the exposed vessels on the inner thigh. Once the arteria femoralis and adjacent vein are filled with contrast agent, the perfusion is complete, and the contrast agent mixture is cured by storing the animals at 4 °C for 24 h.

The tumor area was scanned at 90 kVp, 200 μA with 1000 projections per 180°, integrated for 500 ms with a 35.225 mm  field of view and reconstructed to an isotropic voxel size of 17.2 μm. The scans took 120–240 min depending on how much of the animal was scanned. To facilitate alignment with other modalities, the scans were down sampled to 34.4 μm. The tumor and surrounding tissues were then extracted and placed in a 15-ml centrifuge tube. These were scanned at 90 kVp, 200 μA with 1000 projections per 180°, integrated for 400 ms with 20.48 mm FOV and reconstructed to an isotropic voxel size of 10 μm. Scans of the removed tumor took 105–145 min per sample depending on the tumor size. The resolution of the reconstructed scan can be set depending on the scanning parameters and changes with the field of view and binning. The resolution was chosen to allow a reasonable scanning time given the size of the samples (see Table 3).

#### Post-Processing

**Fig. 2:**
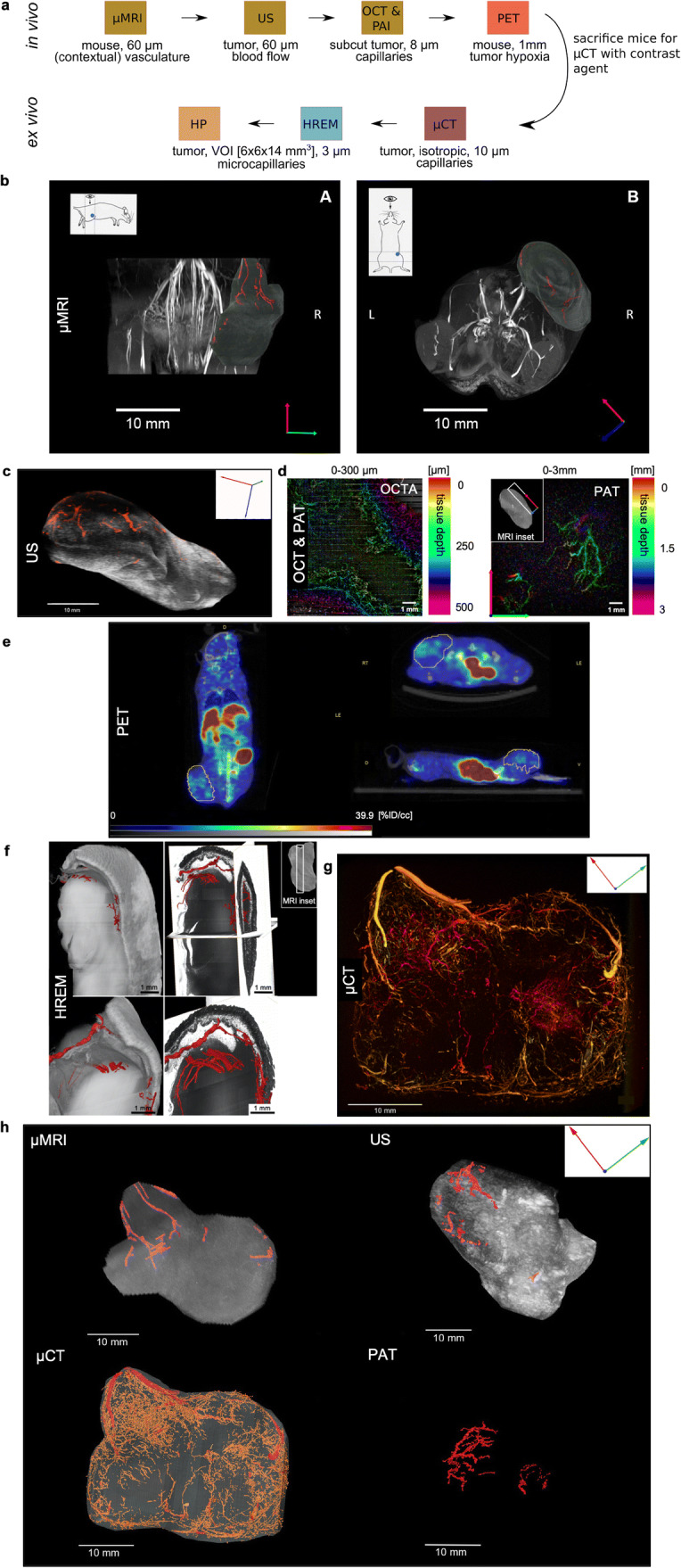
Imaging workflow to visualize multiparametric features of the vascularization of exactly the same murine tumor YUMM 1.7^R^. **a** CMI image acquisition pipeline. The textboxes below the modality indicate the length scale, the achieved lateral resolution, and the measured vascular parameter for each technology. For *in vivo* microMRI, for example, the entire mouse was imaged at an in-plane resolution of 60 μm^2^ and provided insights about the blood vessels along the tumor and the contextual vasculature. For OCT/PAT, only the subcutaneous area of the tumor and corresponding tumoral capillaries were imaged (up to 3 mm in depth) at a resolution of about 8 μm. **b** microMRI of the tumor. Dorsal view with orientation and anatomical context (left), and front view (right). The tumor vessels are indicated in the red channel. Scale bar 10 mm. **c** US, assessing tumor volume and perfusion volume. By using a 3D Power Doppler mode, a rendered 3D image of the tumor with its vasculature (red-coded) was reconstructed. Scale bar 10 mm. **d** OCT/PAT of the same blood vessel network at a lateral resolution of 40 μm with up to 3 mm penetration depth (for PAT). Tissue depth is indicated by the color bar. PAT offers much more detailed insights into the tumor vasculature *in vivo* compared to microMRI but is restricted to subcutaneous areas (right). The image quality of the OCT scan was reduced significantly by heart beating and breathing of the mouse, and only reveals capillaries within a depth range of about 250 μm (left). Scale bars 1 mm.** e** FMISO-PET of the same tumor to assess hypoxia. The microPET scan (jet color scale) is illustrated with co-registered CT data (gray color scale) in three orientations (coronal, sagittal, and transversal slices). The jet color scale indicates the amount of activity in each region as a percentage of the total injected activity (percent injected dose per cubic centimeter [%ID/cc]). The gray scale ranges from − 1000 to 1700 and is measured in Hounsfield Units [HU]. The tumor ROI is delineated in yellow. Increased radioactivity concentrations indicate hypoxic areas and can be found in excretion organs (e.g., liver and intestine). For quantification, tumor radioactivity concentrations were compared to reference soft tissue (e.g., fatty tissue). **f** Segmented blood vessels in an HREM dataset*.* The image shows a combination of volume rendering of the tumor sample and surface rendering of the blood vessels, both based on HREM data. HREM visualizes the microvasculature at an isotropic resolution of 3 μm; the smallest vessels shown here are between 10 and 20 μm in diameter. Vessels were segmented manually. Scale bars 1 mm. **g** Maximum intensity projections of blood vessels in a CT tomogram of the same tumor. The image is a depth cued maximum intensity projection (MIP) of the blood vessels in the Tumor created with Fiji. The colors indicate the tissue depth of the vessels in the viewing direction (depth cue). Compare “Methods” section. Scale bar 10 mm. **h** Representation of all in-toto imaging modalities to visualize the tumor vasculature. Segmented vasculature is shown in red. Due to the orientation of the tumor relative to the scanning probe of the PAT system and due to the limited penetration depth of PAT of only about 3 mm, the big vessels as visualized by microMRI, microCT, and US were not detected by PAT. Scale bars 10 mm.

Reconstructions were performed with the SCANCO microCT software. Reconstructions are stored as ISQ files by the microCT software. For further processing, the scan is exported as a DICOM stack. The final depth cue reconstruction (Fig. 2g) was generated by consecutively creating maximum intensity projections (MIPs) of 5 slice segments and combining these segments into a new stack. This MIP stack was then duplicated for each color channel in an RGB image. Each slice in the MIP stack for the red channel was multiplied by a function which linearly scales the intensity from 75% for the first slice to 100% for the last slice. The MIP stacks for green was linearly scaled from 0% for the first to 75% for the last slice. The color channels were then merged into one RGB stack. From this stack, a MIP was created resulting in the depth cued projection of Fig. 2g.

### HREM

#### Sample Preparation and Data Acquisition

Dissected tumor samples were processed for HREM data generation. Samples were washed in PBS for 2 days and dehydrated in a series of ethanols of increasing concentrations: 24 h in 30%, 50%, and 70% each, 16 h in 80%, 3 h in 90%, and 6 h (two changes) in 100%. The 70%, 80%, 90%, and 100% ethanols contained 0.4 g eosin per 100 ml (Waldeck GmbH & Co. KG, Germany). After dehydration, samples were infiltrated with a JB-4 solutioncontaining 1.25 g catalyst (benzoyl peroxide, plasticized) (JB-4 embedding kit, Polysciences Europe GmbH) and 0.4 g eosin per 100 ml for 7 days (three changes) at 4 °C. Samples were embedded in eosin-dyed JB-4 following a standard embedding protocol and oriented with its proximal end beneath the block surface [23]. For polymerization embedding, molds were sealed airproof for 2 days. Polymerized blocks were baked for 48 h at 80 °C and stored at room temperature until HREM data generation. For data acquisition, the tumor volume was reduced to a VOI as identified by the previous imaging modalities. HREM VOIs comprising approximately 5000 single digital images were generatedautomatically in about 9 h. Data generation was performed following a standard protocol [24, 25]. The resolution is given by the pixel dimensions of each section (2.96 × 2.96 μm^2^) and the section thickness of 3 μm (see Table 3).

#### Post-Processing

Single HREM images are non-specifically contrasted with eosin but permit the identification of larger to smaller blood vessels in combination with the overall tissue architecture of the tumor and the surrounding regions based on the histological appearance of the vessel (Ref: Junqueira L C U and Carneiro J 2003 *Basic Histology: Text & Atlas*: Lange Medical Books, McGraw-Hill, Medical Pub. ISBN 9780071378291). Vascular branches originating from larger vessels were manually traced down to capillary sizes, thus allowing for the identification of blood vessels  that enter the tumor. Volume rendering was used for visualizing the tumor and tissues. Blood vessels were manually segmented with Thermo Scientific Amira Software© while scrolling through the data volume.

### HP

Tumor slices embedded in JB4 resin were sectioned for HREM as described above. Immediately after image capture, sections from selected VOIs were collected in a 24-well plate. The 2D HREM image corresponding to the cut surface of the collected section was noted for subsequent co-registration. The collected JB4 sections were placed onto glass slides and pre-screened by a pathologist. Slides with features of interest were stained by a routine hematoxylin and eosin protocol and subsequently examined with a Zeiss Axioskop 2 MOT microscope. Whole slide digitization was performed with the Pannoramic Flash 250 III scanner (3D Histech). Overview images were acquired from digital scenes with the Case Viewer software (3D Histech). Microscopic images were acquired with a Spot Insight Camera (Diagnostic Instruments).

### Vascular Segmentation

Many imaging systems like Bruker BioSpec, Ettlingen Germany (microMRI) and Vevo2100, VisualSonics, Toronto, Canada (US), have their own visualization software and image format, which cannot be opened by freeware software. These images have to be exported, e.g., as 3D-TIFF or .mhd formats, which can decrease quality. Amira® (V 6.0 Thermo Fisher Scientific) offers a variety of formats to work on, but the quality usually does not match what is visualized in the original software. All 3D datasets were opened in Amira and edited in the Segmentation Editor. Vessels were identified based on their intensity and were segmented by independently thresholding the data from each imaging modality. Automated segmentation had to be improved by manual corrections using the Brush Tool. For microMRI, microCT, US, and PAT, the vessels appeared brighter than the background and were easily identified based on their shape; HREM required substantial manual segmentation. The results were exported as binary images to generate blood vessel masks.

Please note that segmentation, as a prerequisite for the cross-modality co-registration, was not achieved using open-source tools.

**Table 2:** Vascular segmentation and its main challenges for those imaging modalities that provided structural information on the tumor vasculature

Modality	Challenges	Vascular segmentation and visualization	Data format
microMRI	Breathing motion of the mouse	Semi-automated segmentation in Amira (V6.0 Thermo Fisher Scientific) based on thresholding and manual correction	Raw Data as 2dseq; Export into AMIRA as NIfTI
Small-animal US	Motion artifacts of the mouse	Post-processing of 3D data with the in-built Software (Vevo Lab 3.2 0) and subsequent semi-automated segmentation in Amira	Raw Data as VSI; Export into AMIRA as DICOM
OCT/PAT	Vibrations caused by the heart beating and breathing of the mouse	Post-processing by calculating the difference between consecutive B-scans at the same position (OCTA) and detecting the thickness changes of the FPI sensor (PAT) and subsequent semi-automated segmentation in Amira	TIFF
microCT	Quality of perfusion	Semi-automated segmentation using (i) FIJI and AMIRA based on thresholding with manual corrections after visual inspection (*A novel multimodality pipeline*), and (ii) an in-house method based on [26] for the second part (*semi-automated open-source pipeline).* [26] was developed for fully automatic artery segmentation in humans. The generalized version as applied to microCT delivers centerline, vessel radius, and normal information of detected blood vessels in a semi-automated manner from one or several user-defined seed points and directions.	Reconstructions as ISQ files; Export as DICOM
HREM	-Unspecific tissue contrast -Data handling due to large data size	Manual tracing of vessel outlines in image sections in Amira	TIFF

## Results

This proof-of-principle showcase consists of two main parts: (i) demonstrating the feasibility of our CMI approach and the compatibility of 8 imaging modalities from *in vivo* preclinical imaging to *ex vivo* microscopy to sequentially visualize the same melanoma tumor xenograft and its vasculature (CMI image acquisition pipeline), and (ii) co-registering the vascular structure for microMRI, microCT and HREM VOIs of exactly the same tumor with high accuracy (open-source software pipeline). We (i) developed a novel CMI workflow to gather a parameter set that characterizes the vasculature of a single tumor at different length scales including measurements of blood flow and hypoxia (Fig. 2) and allows to compare different mouse models, and (ii) established an open-source software pipeline to co-register the multiscale blood vessel structures across modalities (Fig. 5). All *in vivo* imaging (microMRI, microPET, US, OCT/PAT) was completed within 14 h, followed by the fixation protocol. During this timeframe, we did not expect significant morphological changes of the tumoral vasculature. MicroMRI images were acquired within 1.5 h; the mice recovered overnight for 8 h and were measured sequentially using US (30 min), OCT/PAT (30 min), and microPET (2 h after radiotracer injection for 10 min each). The CMI approach bridges preclinical imaging and *ex vivo* microscopy, spans the relevant resolution range from cm to μm, and characterizes the vasculature *in vivo* in its physiologically active state and at high-resolution *ex vivo* within its structural context. Using CMI, we gradually reduced the imaged and physical volume of the tumor by identifying VOIs and simultaneously increased the lateral resolution. The goal of this study was to establish image acquisition and software CMI pipelines for acquiring images of the same tumor vasculature, and segmenting and co-registering these multimodality data (Figs. 2 and 6). To establish this workflow, we used a melanoma model to illustrate the biomedical benefits and relevance of CMI for selected VOIs only. In the model, we hypothesized that prolonged exposure to targeted therapies not only alters the cancer cells leading them to acquire resistance, but also the tumor microenvironment and, importantly, vasculature. To this end, we utilized the YUMM 1.7 melanoma cell line (*Braf*^V600E/WT^*Cdkn2a*^−/−^*Pten*^−/−^) and a RAFi resistant derivative YUMM 1.7R, which had relapsed after initial tumor regression on continuous administration of RAFi *in vivo* [36]. However, a large array of further studies and functional perturbations will be required to expand on this and deduce novel biomedical insights about the model based on the established pipeline.

In total, 5 mice were injected in each group: 4 of each group were imaged using microMRI, 2 of each group using OCT/PAT and US, 3 of each group using PET/CT. The 4 tumors visualized by all *in vivo* modalities were then imaged *in toto* using *ex vivo* microCT, and selected VOIs of these tumors were further analyzed using HREM and HP.

### A Novel Multimodality Pipeline to Characterize Murine Tumor Vasculature Across Scales

The novel CMI pipeline applied 8 modalities to exactly the same tumor—as can be appreciated in Fig. 2a. Each modality contributes a different length scale and/or parameter to the characterization of the vascular network. US and [^18^F]FMISO microPET contributed blood flow and hypoxia measurements, respectively, and complemented the imaging modalities that typically assess morphology. HP was used for the identification and validation of capillaries and histomorphological features of the tumor.

On the macroscopic scale, we used *in vivo* ultra-high field microMRI at 15.2 Tesla to provide an anatomical reference image with 60 × 60 μm^2^ in-plane resolution and visualize the blood vessels along the tumor and the contextual vasculature of the mouse. In the 3D reconstructed MR images (Fig. 2b), only blood vessels > 100 μm in diameter were clearly discerned in the tumor region (see “Discussion” section).

On an intermediate scale, tumor capillaries down to 10 μm in diameter were visualized by *ex vivo* microCT using a barium sulfate solution as a contrast agent at an isotropic resolution, allowing 3D evaluation of thin vessels which are not visible with other modalities (Fig. 2g). Due to the low attenuation of soft tissue, the higher-intensity values in the microCT scans of soft tissue are caused only by the contrast agent. This makes distinction between vessels and other structures much simpler. The static visualization of the tumor vasculature using microCT was complemented—although at lower resolution—by performing *in vivo* US with a z-resolution of 0.076 mm. With a penetration depth of 15 mm and a lateral resolution of 60 μm, this approach allowed the visualization of the inner tumor vessels (Fig. 2c). Although the vascular network was not resolved at a resolution comparable to microCT, the approach is independent of perfusion and, importantly, in real time. US is specifically useful for the assessment of mature vessels and was used for the identification of VOIs for subsequent microscopic analysis. In addition, US added information about the blood flow within the tumor in addition to the structural information by examining single vessels using pulse-wave Doppler imaging (Table 2 and Supplementary Figure S1). To visualize smaller-sized vessels with a low Doppler signal, we included OCT and PAT—at the expense of penetration depth.

**Fig. 3:**
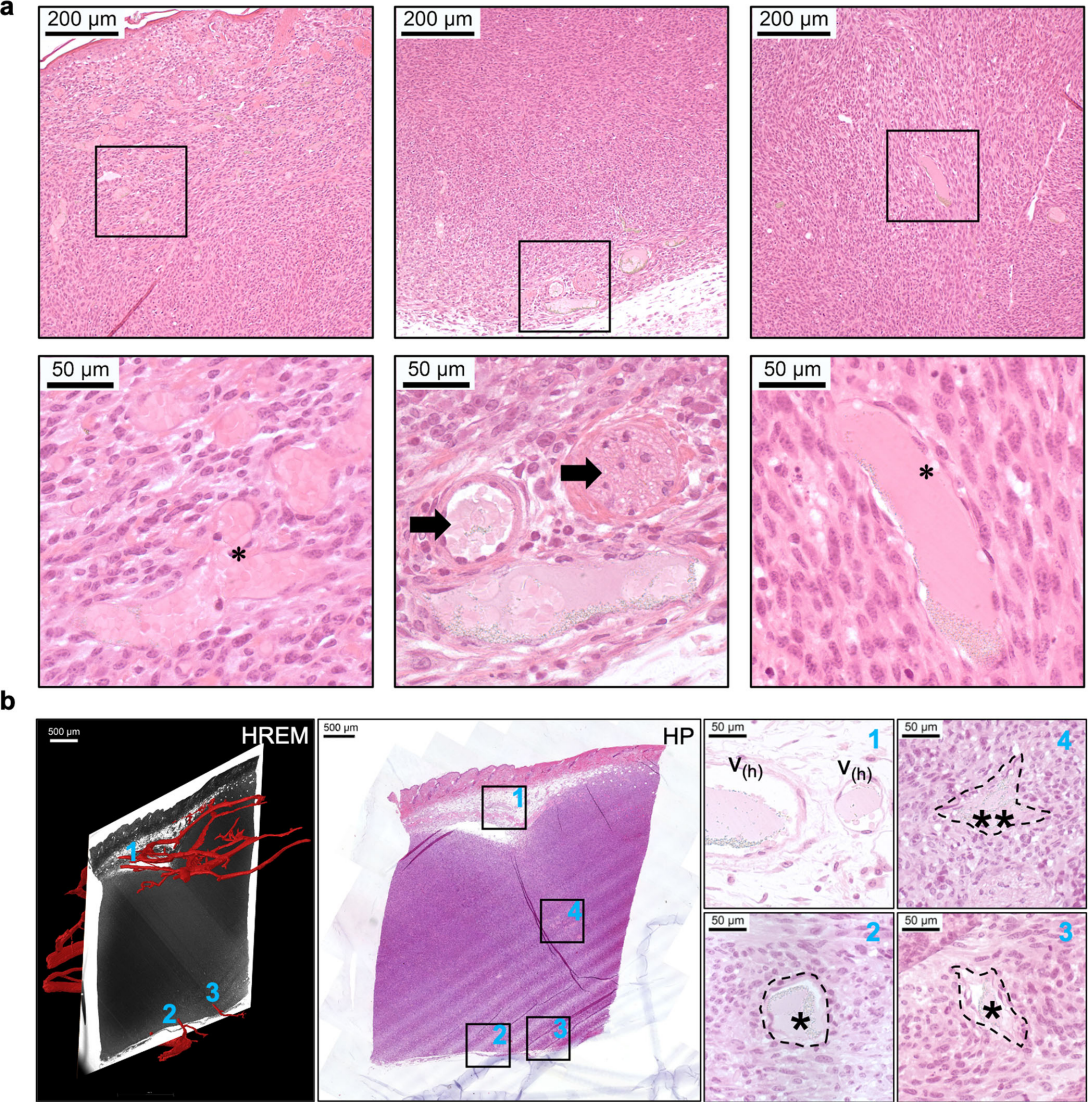
Illustration of HREM-HP co-registration. **a** Representative HP images. Dilated vessels (asterisks), which are frequently congested, are evident within the tumor. Host vessels embedded within the tumor (arrows) are also evident, especially closer to the tumor margins. **b** Illustration of precise regional co-registration between 2D HREM images and HP sections. Representative histomorphological features of a subcutaneous orthotopic melanoma (amelanotic): (1) normal host vessels (V_h_) in the peritumoral subcutaneous adipose tissue; (2, 3, 4) Intratumoral vessels towards the ventral border (single asterisk) and central viable region (double asterisk) of the neoplasm. 1, 2, and 3 represent regions of correlation between HREM and HP

On the microscopic scale, OCT/PAT was used to visualize capillaries *in vivo*, but with a limited penetration depth of about 3 mm. By combining OCT and PAT in one system, the 3D vasculature in the depth range of 0–3 mm was locally resolved non-invasively at the microscale. The combined system assessed morphology (0–500 μm in depth) provided by OCT, vasculature (0–500 μm in depth) provided by OCTA (based on the calculation of the difference between consecutive B-scans at the same position), and vasculature contrast (1–3 mm in depth) provided by PAT at the same time (Fig. 2d). Due to vibrations of the sample due to heart beating and breathing, for the tumor shown in Fig. 2d, OCT quality was significantly reduced and only revealed capillaries locally within an imaging depth of 250 μm. Capillaries deep within the tumor were visualized by HREM. HREM offered the highest resolution of about 3 μm but required sectioning of the tumor. It is a destructive, *ex vivo* block face scanning method, allowing for the automated, rapid generation of highly detailed 3D volume datasets consisting of stacks of inherently aligned images, which can be immediately used for volume rendering and the generation of 3D models. Appearance and image quality of single HREM images is similar to hematoxylin and eosin-stained histological sections. This allowed for identification of blood vessels by using traditional histologic criteria. HREM resolved capillaries in their environment in- and outside the tumor, including the capsule and zone of necrotic tissue, with voxel sizes of 2.96 × 2.96 × 3 μm^3^ within a large-sized VOI of 6 × 6 × 14 mm^3^ as identified by the previous imaging technologies (Fig. 2f). Since blood vessels do not need to be perfused or selectively contrasted for 3D visualization, blindly terminating or beginning vessels, short collaterals inside the tumor and its necrotic parts were clearly visualized. Following manual segmentation and 3D surface modeling, the tissue architecture was analyzed. Since this proved to be very time-consuming (35 h for 5000 sections), 3D modeling was restricted to selected VOIs. Since, except for resin embedding, no special chemicals are required for tissue processing, HREM can be easily combined with almost all up- and downstream techniques of the established imaging pipelines. It proved to be the method of choice to reconstruct tumor capillaries of sufficient contrast and quality at micrometer resolution in selected VOIs. We showed that HREM is compatible with contrast agent-enhanced CT and with the fixation, staining, and dehydration media used after tumor removal for microCT and HP (Fig. 2f). Even though HREM is destructive to the tissue, we collected physical sections for subsequent histopathologic examination. All HREM results were validated by HP based on conventional H&E stained sections (Fig. 3).

Further information about hypoxia within the tumor was added by microPET (Fig. 2e), using [^18^F]-fluoromisonidazole ([^18^F]FMISO) as the most commonly used PET tracer to image the lack of oxygen [8, 37]. PET allows the quantification of the uptake process and, hence, the assessment of the extent of hypoxia. Since it is difficult to morphologically resolve and spatially localize the PET signal, it is important to combine the technique with microCT to guide the spatial alignment of the tumor. Due to the limited resolution of PET, only the combination of PET with other imaging technologies allowed us to put the information about hypoxia into its structural context. Each animal was imaged on the same instrument using microCT, which was co-registered to the microPET image. Matching anatomical landmarks were used to identify the 3D VOIs. To allow comparison of different animals, radioactivity concentrations were expressed as SUV (g/mL). A trend towards lower blood flow and elevated hypoxia was observed from the initial microPET and US measurements for RAFi resistant YUMM 1.7^R^ tumors. However, the limited number of animals and noisy *in vivo* data precluded a more detailed analysis of these parameters (see below, Table 3).

**Table 3:** Validation and comparison of characteristics of the vasculature as measured by different modalities. The structural parameters are extracted from a single tumor (compare Fig. 2) and are compared across 5 different modalities. Hypoxia and blood flow are averaged values from 2 to 3 animals that were imaged per group by a single modality. Note that microCT data were segmented automatically, resulting in more fragmented blood vessels

	**microPET**		**US**		
	***Hypoxia [SUV]***		***Blood flow [mm/s]***		
Yumm 1.7^R^	1.9 ± 1.8		14 ± 1.4		
Yumm 1.7	0.3 ± 0.3		23.5 ± 2.1		
	**US**	**microMRI**	**microCT**	**PAT**	**HREM**
	***Resolution [mm]***				
All	0.037 × 0.037 × 0.076	0.06 × 0.06 × 0.2	0.034^3^	0.034^3^	0.003^3^
	***Tumor volume [mm***^***3***^***]***				
1.7^R^	1099	922	990	/	3.14*
1.7	1128	824	931	1518**	5.004*
	***Vessel volume [mm***^***3***^***]***				
1.7^R^	1.66	0.25	7.13	/	0.02*
1.7	4.23	1.45	12.25	1.13	0.062*
	***Perfusion volume [%]***				
1.7^R^	0.8	0.03	0.72	/	0.64*
1.7	1.5	0.18	1.32	0.074**	1.24*
	***Total vessel length [mm]***				
1.7^R^	35	7	668	/	18*
1.7	78	45	1433	60.13	34*
	***Number of segments***				
1.7^R^	50	8	1321	/	174
1.7	107	30	4181	109	125
	***Longest segment [mm]***				
1.7^R^	2.6	1.3	4	/	1.2
1.7	5.1	5.8	4.3	3	3.33
	***Mean length [mm]***				
1.7^R^	0.6	0.9	0.5	/	0.11
1.7	0.5	1.4	0.5	0.6	0.27

*Refers only to the segmented VOI of the tumor; fraction of entire tumor

**Tumor not visible; refers to the entire volume of the acquired image

Together, microMRI, US, microCT, PAT, and HREM created a detailed 3D map of the tumor vasculature at high-resolution in VOIs (Fig. 2h).

Practical aspects for the setup of our multimodality workflow to facilitate data co-registration are highlighted in the following: the CMI workflow required substantial coordination, careful sample preparation procedures that were compatible across all modalities without compromising data quality (including the sequence of experiments, sample thickness and fixation, and staining protocols), and strategies to determine the orientation of the tumor and relocate the VOIs across imaging platforms. For *in vivo* imaging, the same tumor needed to be imaged sequentially as fast as possible to rule out significant structural changes at the micro- and macro-scales between relocations. All mice were imaged *in vivo* within 14 h using microMRI, US, OCT/PAT, and microPET. Since all animals were imaged sequentially using several *in vivo* imaging modalities, it was decided not to administer any US contrast agent to keep the overall burden as low as possible. The used MS550S probe (center frequency 32 MHz, maximum frequency 55 MHz) is well suited for Color and Power Doppler imaging and also anatomical imaging. The switch to a probe with a higher frequency (70 MHz) would have been possible to enable greater spatial resolution without compromising depth for a tumor of this size, but we decided to keep the acquisition time for each technology minimal to facilitate co-registration of vascular data. During the OCT/PAT experiment, rectangular shaped black tape on the top surface of the tissue was applied, is visualized both by OCT and PAT at the same time and used for co-registration of the modalities (and can be used for the co-registration with other modalities as well). Usually, vasculature in the depth range of 300–500 μm can be visualized by both OCT and PAT and can also be used for co-registration. MicroPET was carried out as the last *in vivo* modality to allow radioisotope decay after fixation. To our knowledge, this is the first instance of the integration of microPET into such an extensive CMI pipeline. Mice were anesthetized, imaged by microPET, perfused with a vascular microCT contrast agent, and sacrificed. Tumors were isolated and fixed with adequate adjacent normal skin and soft tissue margins to facilitate co-registration across modalities. The microCT contrast agent did not interfere with HREM and HP characterization. For *ex vivo* imaging (i.e., microCT, HREM, and HP), identification of the VOI along with its orientation was crucial—which was facilitated by including peritumoral tissues and utilizing adjacent anatomic landmarks to maintain matched orientations in sections. To that end, notches were placed on the skin to correspond with the cranial, caudal, medial and lateral boundaries of the tumor. The orientation of the dissected tumor samples was noted when the tissue was embedded in JB4 resin for HREM. The proximal aspect was at the top of the JB4 block and corresponded with the skin side of the tissue. The distal aspect was at the base of the JB4 block and corresponded to the deeper tumor tissue. Two-dimensional HREM images were acquired and three dimensionally stacked in a proximal to distal sequence. Identification of the skin surface and preparation of proximal to distal image stacks allowed recording of the position of the HREM volume in respect to the tumor. JB4 resin sections were collected during HREM data acquisition and further studied by HP to validate the tumoral capillaries that were segmented based on HREM datasets. Registration was achieved between H&E-stained resin sections (collected post-HREM) and HREM images by matched identification of the skin, subcutaneous vessels, nerve bundles, and mammary duct. Examples of normative and neoplastic vascular features that can be discerned at light microscopic resolution by HP are illustrated in Fig. 3a. Examples of HREM and HP data co-registration are illustrated in Fig. 3b.

For the comparison of cancer cells with and without acquired resistance, we quantified a set of structural parameters to characterize the vasculature of one tumor each from the parental YUMM 1.7 and RAFi-resistant YUMM 1.7^R^ groups. The CMI approach also allowed the comparison of extracted parameters across modalities for exactly the same tumor (Table 3). Since the chosen modalities were complementary, it is not surprising that the quantification of the vascular data varied greatly between the modalities since each technique revealed different tumor vessels at different length scales and penetration depths. Comparison of the cross-modality values must be done with caution since the modalities complement rather than validate each other. Even the measurements of the tumor volume differed between the modalities by up to 35% (e.g., 1128 mm^3^ as measured by US versus 824 mm^3^ by MRI). PAT did not unambiguously delineate the tumor. Since HREM of an entire tumor is tedious and time-consuming, a limited VOI within the tumor of about 5 mm^3^ was selected as identified previously by microCT and only the detected vessels within this VOI were analyzed using HREM. Apart from hypoxia and blood flow, from the set of quantified structural parameters, only the perfusion volume as a ratio (vessel volume to total tumor volume) and the mean vessel length as an average value allow comparability between tumors due to the differences in resolution and hence detected vessel lengths and volumes across modalities. While the values of the perfusion volume and the mean lengths of the YUMM 1.7^R^ tumor varied substantially across modalities (from 0.03 to 0.8 mm^3^ and from 0.11 to 0.9 mm), there is a consistent tendency in the data across all modalities when comparing YUMM 1.7 with YUMM 1.7^R^ tumors: The tumor derived from the parental cell line showed higher perfusion volumes across all modalities and longer vessels on average (both the longest segments and mean lengths). In addition, the blood flow was higher and the tumor was less hypoxic. The RAFi resistant YUMM 1.7^R^ vasculature seemed to be more fragmented with much smaller longest segments and on average smaller mean lengths across all modalities (with the exception of US), and more dysfunctional as indicated by higher hypoxia values and lower blood flows.

### Semi-automated Open-Source Pipeline for the Co-registration, Visualization, and Interpretation of Multiscale Vascular Imaging Data

**Fig. 4:**
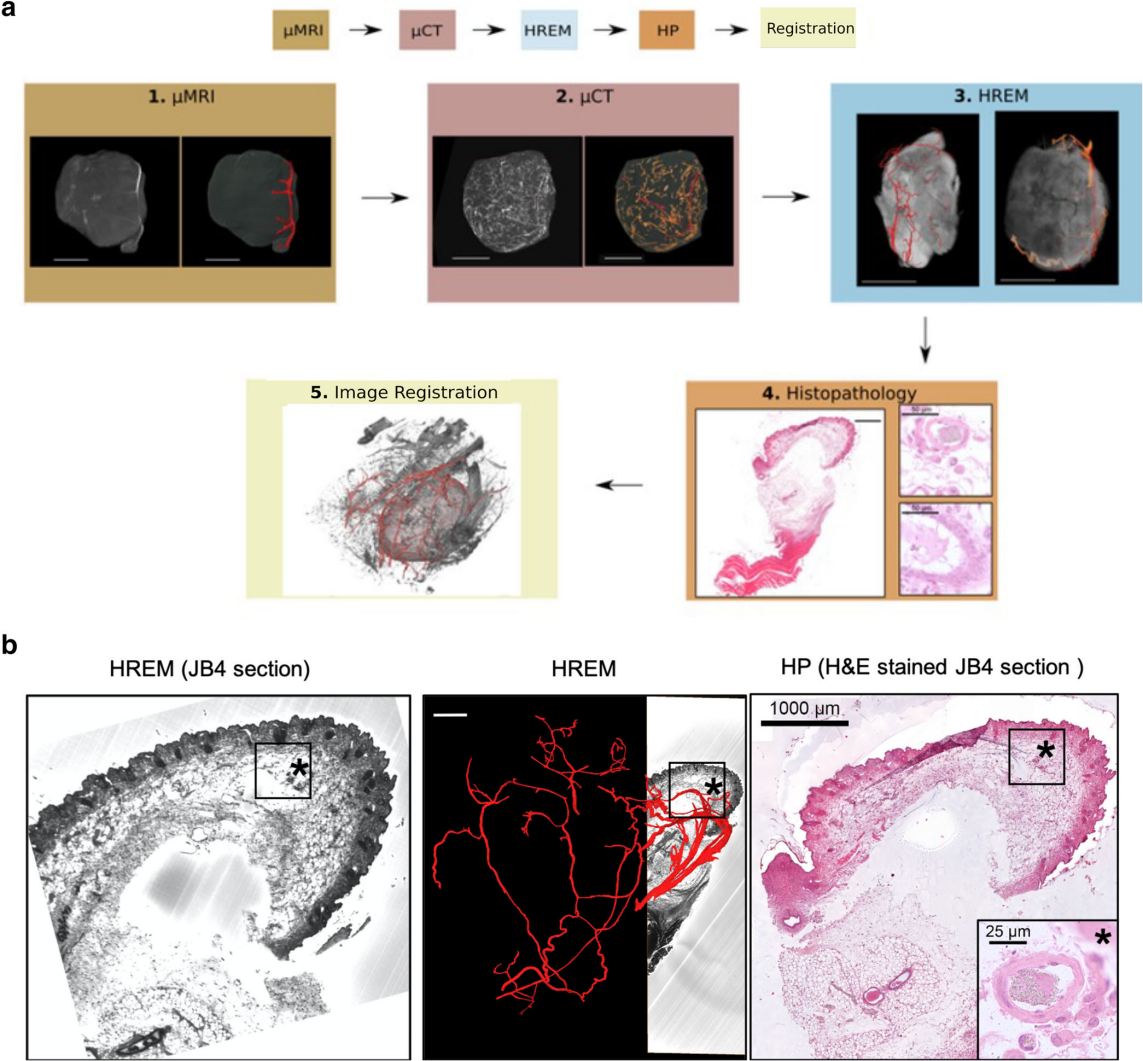
**a ***In vivo* and *ex vivo* imaging workflow to visualize the blood vessels of exactly the same tumor at various scales for a selected microMRI, microCT, and HREM dataset. a1, left shows the isolated tumor as acquired by microMRI and shown as maximum intensity projection (MPI); the right side indicates the MIP with segmented vessels, which are shown in red. a2, left shows the isolated tumor acquired using microCT; the right side depicts the MIP with already segmented vessels. The red channel indicates the same vessels as in microMRI, the orange channel shows smaller vessels that were not detected by microMRI. a3: Due to the limited VOI that can be acquired using HREM, the tumor had to be cut in 2 halves for HREM data generation. Each half is shown in a3 (left and right side) with segmented vessels in red. a4 depicts a histological slide of the tumor. HP was used to confirm segmented blood vessels based on the HREM data. a5 shows the co-registration of the microMRI, microCT and HREM vascular data for this tumor in red. Anatomical context is provided by microCT. **b** Co-registration of HREM and HP datasets for the validation of capillaries as segmented by HREM. The co-registration is achieved for the same tumor as shown in Fig. 5. Tumor slices embedded in JB4 resin were sectioned during HREM data acquisition as described above. Immediately after image capture, sections from selected VOIs were collected for further HP processing. Sections with features of interest were stained by a routine hematoxylin and eosin protocol. Blood vessels were segmented manually on selected HREM sections using AMIRA. Since the eosin contrast used for HREM is unspecific for vascular contrast, the manual vascular HREM segmentation was confirmed and validated on the same section by HP. Sections were matched manually

Apart from developing an advanced image acquisition workflow to generate a multiscale parameter cloud for the comprehensive characterization of tumoral vasculature, we also established the corresponding software pipeline to co-register selected structural data (Fig. 4a). We set this pipeline up such that it is both freely available (open source) and as automated as possible. In the following, we describe and showcase the established software workflow for 3 selected multiscale datasets from microMRI, microCT, and HREM from *in vivo* imaging at 60 μm to *ex vivo* microscopy of a few μm. As before, HP was used to validate capillaries identified by HREM (Fig. 4b).

**Fig. 5:**
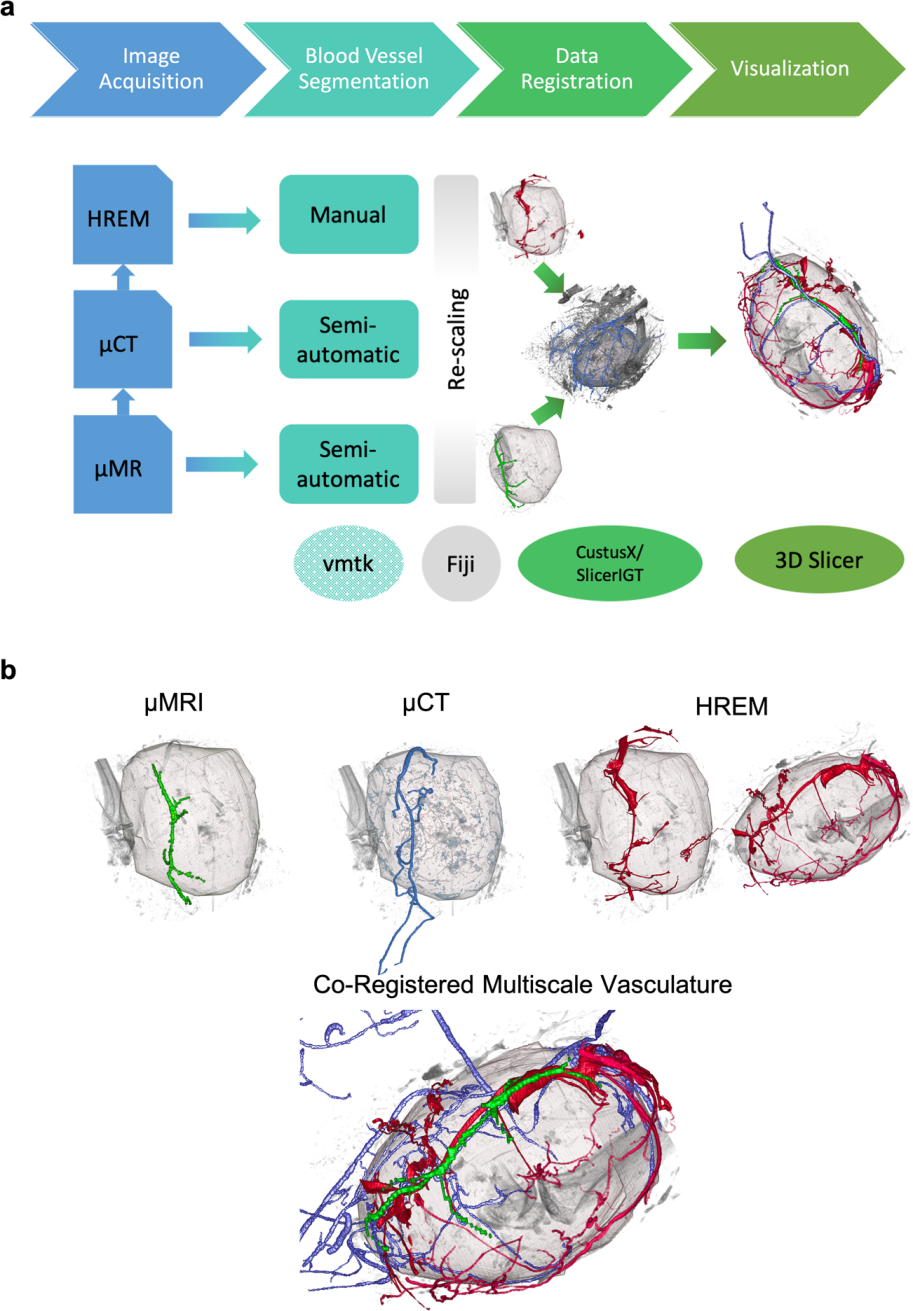
**a** Semi-automated open-source software pipeline for the visualization of the co-registered vascular tumor network across scales. Please note that open source refers to the data co-registration and visualization parts only as discussed in the text. For the segmentation of blood vessels, we used the commercial software AMIRA, but suggest VMTK as an open-source alternative (http://www.vmtk.org/). After vascular segmentation, the open-source pipeline includes re-scaling using ImageJ2/FIJI [27–29], data co-registration using CustusX [30, 31] and SlicerIGT [32, 33], and visualization using 3D Slicer [34, 35]. We showcase this software pipeline for the co-registration of vasculature based on microCT, microMRI, and HREM data of the same tumor. **b** Co-registration of the vasculature as segmented based on microMRI, microCT, and HREM datasets. Segmented tumoral vasculature as described in detail in the text is depicted in red for each of the modalities for exactly the same tumor. HREM shows the tumor from two different perspectives. The segmented vascular datasets are co-registered and integrated to visualize the multiscale vasculature of the entire tumor across scales. For the co-registered vasculature, the red channel indicates vessels segmented based on HREM datasets, green shows microMRI, and blue represents microCT vascular data

**Fig. 6:**
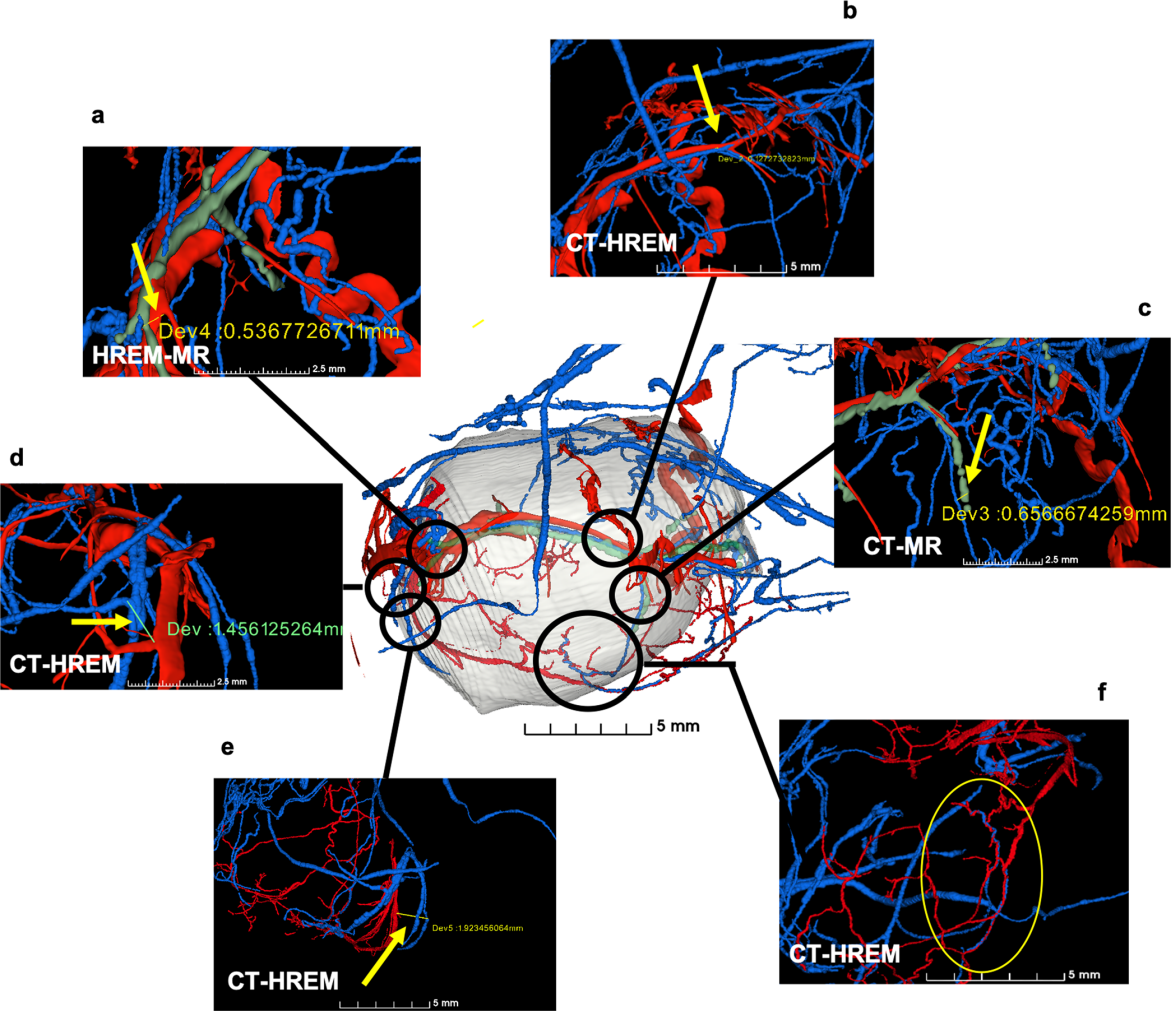
Co-registration quality. Manual measurements illustrating the co-registration quality achieved using affine registration based on manual landmarks using SlicerIGT on vascular data segmented on microCT (blue channel), microMRI (green) and HREM (red) data. Compare with Fig. 5b. Deviation in **a** of co-registered HREM and microMRI vessels is about 500 μm, in **b** about 100 μm between microCT and HREM, and in **c** about 650 μm between microCT and microMRI co-registrations. In the lower part of the tumor, microCT and HREM vessels are almost perfectly matching locally as highlighted by the yellow circle (**f**) but deviate in the millimeter range (deviation in **e** about 2 mm) at the border of the HREM sample due to deformations induced during HREM data acquisition due to the sectioning of the tumor (**d**, **e**)

The established semi-automated pipeline for data co-registration consists of three major steps: blood vessel segmentation, data co-registration, and visualization. An overview of the pipeline is illustrated in Fig. 5a. For blood vessel segmentation, there is currently no open-source vessel tracker available, general enough to handle all imaging modalities. Therefore, blood vessels visualized using microMRI, microCT, and HREM were segmented using Amira; segmentation of microCT for the tumor shown in Figs. 5 and 6 was additionally achieved using a semi-automated vessel tracker based on [26]. As an open-source alternative, the Vascular Modeling Toolkit (VMTK) (http://www.vmtk.org/) [38] is proposed. Co-registration and visualization were accomplished using available open-source software. MicroCT was chosen as the joint reference space for the co-registration as it establishes a trade-off between a data size feasible for the tools used in this pipeline (i.e., the need to co-register and visualize the data on a standard PC) and a spatial resolution providing enough detail for a comparison of the segmented blood vessels. For compatibility purposes, all data were converted to .mhd format using the export functionality of ImageJ2/FIJI [27–29]. Spatial correspondence was established using pairwise affine landmark-based registration. To accelerate image registration and to reduce the memory footprint in particular of the HREM data during the registration process, the plugin “Resample Scalar Volume” of 3D Slicer [34, 35] or alternatively ImageJ2/FIJI can be used to resample the segmented blood vessel masks derived from the HREM and microMRI images to microCT resolution. To generate blood vessels masks for co-registration, segmented vessels from the different modality datasets were exported as binary images in Amira.

To correctly identify corresponding landmarks on the blood vessel trees, spatial orientation is crucial and landmark-setting in 3D view is critical. CustusX [30, 31] and the 3D Slicer plugin SlicerIGT (Ungi et at 2016; http://www.slicerigt.org) are open-source tools for image-guided intervention offering pairwise affine landmark definition directly in the 3D view with the possibility of fine-adjustment in co-axial 2D slice views. Using these tools, corresponding branching points were identified on blood vessel masks from microCT, microMRI and HREM and used for spatial alignment, where SlicerIGT provided better interaction possibilities for precise landmark placing. Aligned mask volumes including the transformation matrices were stored as *.*mhd files. The multimodality volume-rendering functionality of 3D Slicer allowed to visualize all data together as illustrated in Figs. 5b and 6. Alternatively, tomviz (www.tomviz.org) [39] can be used but requires previous resampling of the aligned blood vessel masks to the microCT grid as CustusX and SlicerIGT store only transformation matrices as part of the *.*mhd files but do not automatically resample all data to the reference space. The tumor outline, manually segmented based on microMRI data, provides the context for the visualized blood vessels in 3D Slicer. This illustration provides detailed information on the tumor vasculature and allows the comparison of the blood vessels detected by the different modalities and a validation of the segmentation across modalities for those vessel segments identified in more than one modality. The visualization reveals remaining deformations after affine registration in particular in respect to HREM data (Fig. 6d, e). This is not surprising due to HREM’s serial block-face acquisition scheme, which continuously slices the sample and leads to distortions. Matching of blood vessels was possible for microCT, microMRI, and in the upper part also for HREM with submillimeter accuracy (Fig. 6a–c). In the lower part of the tumor, no blood vessel segmentations of microMRI were available. Affine matching of microCT and HREM data was achieved on a local level at high accuracy (Fig. 6e), but not globally (Fig. 6d, e) as the HREM sample of this part of the tumor was heavily deformed in a non-linear manner during sample preparation and data acquisition. Quality of the co-registration was confirmed via visual inspection (Fig. 6a–f).

## Discussion

The chosen imaging modalities complemented and validated each other and helped to overcome limitations that are faced when stand-alone techniques are used:

For microMRI, for example, we estimate that only blood vessels > 100 μm in diameter were clearly visualized in the tumor region. This suggests that smaller blood vessels were difficult to visualize due to a slower blood flow compared to normal vasculature. This hypothesis is supported (i) by the visualization of smaller vessels in the abdomen, while such vessels were not visualized inside the tumor (Fig. 2b), and (ii) by the low blood flow of tumor capillaries determined by US. In addition, based on the established CMI pipeline including higher-resolution techniques, we confirmed that tumor blood vessels have cross-sections smaller than the in-plane resolution of the scan (~ 60^2^ μm^2^), making the visualization difficult due to the partial volume effect. Using microMRI alone, we could not resolve the tumor vasculature and not establish whether the poor visualization of tumor vasculature was due to small blood vessels or reduced blood flow in these vessels or both. For microCT, a complete perfusion with the contrast agent was difficult to achieve and there could always be small vessels that were not reached by the contrast agent and consequently invisible in the microCT scan. Overall, two of the four tumors were insufficiently perfused. Even with perfect perfusion, the soft tissue contrast in the microCT is poor, allowing only a rudimentary distinction between different tissues without any fine detail. MicroCT further provided no functional information and proper oxygen supply can only be inferred from the presence or absence of vessels. To overcome these limitations and artifacts, the visualization of the tumor vasculature needs to be complemented by the other modalities used in this proof-of-concept study.

However, despite its potential to assess tissue vasculature holistically across scales, the established CMI workflow presents several challenges from a technological point of view. Due to the many technologies involved, the pipeline is expensive and time-consuming. It requires a broad range of expertise across different imaging modalities, careful coordination, thoughtful sample preparation procedures, and correlative markers. We find two major bottlenecks for the routine implementation of these extended CMI pipelines in the future: (1) availability of research infrastructure (i.e., cutting-edge technologies from microMRI to advanced microscopy), and (2) data handling and co-registration of the different imaging datasets.

1. There are several initiatives that facilitate access to, and integration of, complementary imaging technologies [40]. The most prominent one might be Euro-BioImaging, a research infrastructure consortium that offers open access to a vast repertoire of biological and biomedical imaging technologies. Euro-BioImaging consists of imaging facilities, called nodes that have opened their doors to all life science researchers.
2. Many solutions have been proposed for blood vessel segmentation [41], reconstruction [K. Bühler, P. Felkel, and A. La Cruz, “Geometric methods for vessel visualization and quantification—a survey,” in Geometric Modeling for Scientific Visualization, G. Brunnette, B. Hamann, H. Müller, and L. Linsen, Eds. Springer, 2004, pp. 399–419.] and vascular image registration [S. Matl, R. Brosig, M. Baust, N. Navab, and S. Demirci, “Vascular image registration techniques: A living review,” Med Image Anal, vol. 35, no. C, pp. 1–17, January 2017]. The importance of visual representations of imaging data has already been highlighted by Walter et al in 2010 [42] and many of the software tools listed there have been further developed in the past years and were also used for our feasibility study. The visualization of multimodal data poses an additional conceptional and technical challenge, which has been discussed recently by Lawonn et al. [43]

However, established software workflows that are generally applicable across different modalities to correlate vascular imaging data have been missing. With the showcased pipeline, we present a quick general solution for any biomedical scientist to co-register vascular data, but also demonstrate the limitations and open problems when dealing with vascular data from several modalities. This workflow faces a general challenge in image analysis and quantitative biology.

While prior segmentation of blood vessels is required for a reliable co-registration of datasets in the absence of fiducial markers, automated segmentation can only be achieved in a few cases, due to low signal-to-noise ratios (SNRs) and different contrast mechanisms across modalities. Even though intensity thresholding and binary morphological operations identified the majority of blood vessels, the correct assignment has to be reviewed by an experienced user. Filtering, thresholding, and more advanced blood vessel tracking [46] operations succeeded (partially) for microMRI, OCT/PAT and microCT, but failed for HREM. These datasets had to be segmented manually with all its challenges, presenting a major bottleneck in the co-registration workflow as it is time-consuming and partially subjective. Main challenges for the vascular segmentation for each (structural) modality are summarized in Table 2. For the *in vivo* imaging technologies, motion artifacts, for example due to breathing of the mouse, reduce the image quality and, hence, the quality of the vascular segmentation. For US, acquisitions in Power Doppler Mode are prone to motion artifacts although we used respiration gating during the acquisition. For OCT and PAT, the main factor that decreased the image quality was the motion of the tumor caused by the heart beating and breathing of the mouse. To improve vascular segmentation and co-registration of datasets, data acquisition time needs to be minimized and a motion correction algorithm to be developed. Currently, the bottleneck of the data acquisition time of the PAT system is the low repetition rate (100 Hz) of the pulsed excitation laser. We are developing multi-beam detection algorithms to minimize the data acquisition time significantly. For microCT, the main challenge is the quality of the perfusion as described above, which can only be assessed after image reconstructions. In addition, at 10 μm resolution, only blood vessels larger than 20 μm can be segmented reliably (as they become too dark to be distinguished from the background noise). For HREM, vascular segmentation is most challenging and usually needs to be performed fully manually due to the unspecific contrast of the blood vessels in the HREM datasets. Manual segmentation is time-consuming and—due to the large size of one HREM dataset of several GB due to its micrometer resolution and capability of visualizing relatively big volumes of several mm^3^—not trivial to handle in terms of computing power and visualization.

In addition, due to the different fixation and data acquisition protocols for the *ex vivo* modalities, distortions of the tissue vasculature of several microns are introduced and present another challenge. Remaining deformations, which can be tackled with non-rigid registration, are an open issue as reliable landmark-setting along incomplete vessels is not possible. Furthermore, deformable registration influences morphological parameters of the blood vessels and is not feasible if the morphological analysis of the vasculature is subject of the study (as in our case). However, deformable registration will be required to achieve a full matching of the different modalities. The identification and matching of corresponding landmarks along incomplete and heavily deformed vessels to guide a non-rigid registration process is extremely challenging even for experts. Currently, there are no tools available to support such a workflow and/or semi- or even fully automated registration task. In our experience, manual segmentation and visual inspection currently present the only way to co-register distorted imaging datasets of low SNR. Using manually segmented vessels as landmarks, we were able to co-register capillaries from diverse datasets locally as revealed by visual inspection, which allowed the identification and assessment of corresponding blood vessels across modalities. In general, high-quality segmentation and correlation of blood vessels requires dedicated vessel segmentation software like AMIRA, vmtk [38], or [26] across all modalities.

If the images are acquired across several labs using different software, imaging, and data protocols, an additional source of data incompatibility is likely to be added. Since data formats in bioimaging are diverse, careful planning and joint agreement on formats for annotation and data before the project start can save tremendous amount of time when it comes to data fusion and visualization. This not only includes the storage mode of a dataset, but also its color/intensity value depth. Another important point is an agreement on how annotated structures should be stored (each segmented structure in a single file or several masks in one file; binary masks or geometrical arrays) and to agree on naming conventions (clear naming conventions for biological structures of the same type; same name for the same structure across different images if correspondences are already known). The final choice of formats is in the best case aligned to the software pipeline and the hardware resources to handle the data. For our open-source pipeline, we stored all data as 8-bit values and used the MetaImage format *.*mhd, accepted by many open-source image processing tools.

The choice of the reference space of a stack of correlated images is another important factor in vascular data co-registration and generally defines the final resolution of the result. If joint visualization is the goal as in our case, the choice of the reference space should reflect the best trade-off between showing enough context and revealing all relevant information at the best possible resolution across all modalities. If the range of spatial scales to be fused is too large to map all relevant information to a joint resolution at the required detail, it might be beneficial to align the data to several reference spaces at different resolutions and to create several overlapping/complementary visualizations. Another vital point to be considered when choosing a reference space is its final demand on hardware and software requirements. For our CMI pipeline, microCT proved to be the best (trade-off) reference space.

The choice of software for our novel co-registration pipeline was well aligned with the requirements set by the imaging data to be fused. Image-alignment software (CustusX, Slicer IGT) was capable of co-registering the data in a reasonable time, considering the size of the chosen reference space and input images. Although fiduciary markers visible across all imaging modalities were not available in our case, manually placing and identifying corresponding markers in 3D accelerated the co-registration tremendously. The size and type of the data to be rendered at once influenced the choice of the visualization software. Special software (3D Slicer, tomviz) for out-of-core data visualization was required as the size of the data exceeded the CPU and GPU RAM of the visualization workstation. Not all volume-rendering tools are capable of fusing several volume datasets at once or correctly fusing geometry and volumetric data.

In future work, a software tool including automated segmentation, co-registration, and visualization has to be developed to help automating the established workflow. In this software, a solution for vessel segmentation across different modalities can be incorporated to provide an end-to-end solution for the entire workflow.

With this novel CMI pipeline, we help to nurture the continued discovery in tumor vascularization and the development of imaging approaches. The combination of complementary technologies that are sensitive to vasculature at different scales, physiological states and underlying tissue architecture will contribute to the understanding of the interplay between vascular macro- and microstructure, blood flow, and hypoxia. Since a multitude of datasets need to be taken and analyzed to obtain statistical conclusions, it is extremely time-consuming to define the relationships between the diverse parameters visualized using CMI pipelines. In future, based on our CMI approach and the parameter cloud acquired from a single tumor, it will therefore be desirable to implement machine learning algorithms to facilitate the classification of tumors [44]. From a biomedical point of view, complementary multimodality imaging of the tumor vasculature should be included as a vital part in cancer management plans [45].

## Conclusion

The presented work focuses on the establishment and demonstration of a multimodality imaging pipeline to characterize the tumor vasculature across scales. While in this proof-of-principle study the animal experiments were not powered to identify quantitative differences, it paves the way for studies to understand disease mechanisms (e.g., how tumor vasculature influences metastasis) or to identify therapeutic entry-points that make tumors responsive to therapies (e.g., by understanding how targeted therapies that inhibit the oncogenic signaling pathways alter tumor vasculature or how to enhance T cell infiltration and make tumors amenable to checkpoint inhibitors). The visualization of the vascular network across scales set the basis for the implementation of a novel workflow of blood vessel co-registration to resolve and validate the vascular network of an entire tumor and its surroundings. Blood vessels segmented from several modalities were co-registered using microCT as reference space. The pipeline is accessible to any researcher interested in the multiscale organization of tissue and will enable insights into how tumors instruct the formation and maintenance of blood vessels in response to oncogenic perturbations and pharmacologic interventions. In contrast to most of the previously performed multimodality studies, we succeeded in establishing a truly correlative imaging approach from *in vivo* to *ex vivo* that was carried out on the very same specimen across modalities. We employed semi-automated open-source tools for co-registering images from different modalities. The CMI image acquisition and software pipeline provides a versatile tool to inter-relate vascular function and structure of the same tumor and will facilitate the integration of multiscale and multimodal vascular datasets in preclinical cancer models. It represents a highly needed cooperative and interdisciplinary approach that brings together radiochemistry, 3D *in vivo* and *ex vivo* imaging of high sensitivity and high resolution, and optimized image co-registration workflows. Although focused on vascular imaging, the established CMI pipeline will be beneficial for the detailed and holistic characterization of several important aspects of tumor biology, including the tumor microenvironment. Beyond the fusion of multiscale structural and functional imaging, our multiparametric workflow will allow correlative analysis of the interconnected hallmarks of cancer and better characterization of the status of a single tumor.

## Supplementary Information

ESM 1 (PDF 255 kb)
