# Supplementary material for: Cross-Modality Imaging of Murine Tumor Vasculature—a Feasibility Study
Source: Mol Imaging Biol. 2021 Jun 8;23(6):874–93. doi: 10.1007/s11307-021-01615-y (PMC8578087; doi:10.1007/s11307-021-01615-y)
Supplement: Supplementary file 1 — (PDF 255 kb) [file 11307_2021_1615_MOESM1_ESM.pdf]

Supplementary

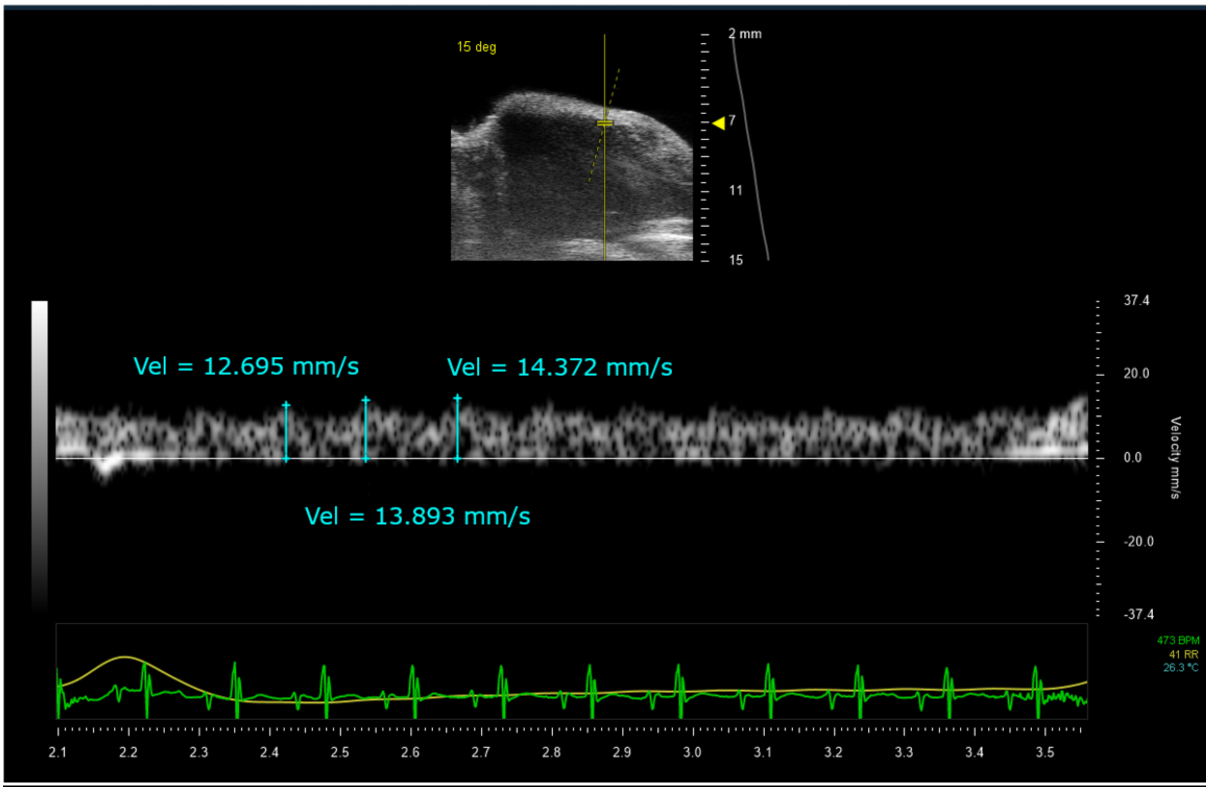

Figure S1. *Illustration of the slow blood flow in tumoral vasculature for the same tumor as shown in Fig. 2, using Doppler Imaging.*
